# Preparation of High-Toughness Lignin Phenolic Resin Biomaterials Based via Polybutylene Succinate Molecular Intercalation

**DOI:** 10.3390/ijms24076418

**Published:** 2023-03-29

**Authors:** Jin Xie, Hao Sun, Yuchun Yang, Junxiong Liang, Yun Li, Defa Hou, Xu Lin, Jun Zhang, Zhengjun Shi, Can Liu

**Affiliations:** 1 International Joint Research Center for Biomass Materials, Southwest Forestry University, Kunming 650224, China; 2 Graduate School of Agriculture, Kyoto University, Kyoto 606-8502, Japan

**Keywords:** polybutylene succinate, intercalation method, toughening, lignin, biomaterials

## Abstract

Lignin has many potential applications and is a biopolymer with a three-dimensional network structure. It is composed of three phenylpropane units, p-hydroxyphenyl, guaiacyl, and syringyl, connected by ether bonds and carbon–carbon bonds, and it contains a large number of phenol or aldehyde structural units, resulting in complex lignin structures. This limits the application of lignin. To expand the application range of lignin, we prepared lignin thermoplastic phenolic resins (LPRs) by using lignin instead of phenol; these LPRs had molecular weights of up to 1917 g/mol, a molecular weight distribution of 1.451, and an *O*/*P* value of up to 2.73. Due to the complex structure of the lignin, the synthetic lignin thermoplastic phenolic resins were not very tough, which greatly affected the performance of the material. If the lignin phenolic resins were toughened, their application range would be substantially expanded. Polybutylene succinate (PBS) has excellent processability and excellent mechanical properties. The toughening effects of different PBS contents in the LPRs were investigated. PBS was found to be compatible with the LPRs, and the flexible chain segments of the small PBS molecules were embedded in the molecular chain segments of the LPRs, thus reducing the crystallinities of the LPRs. The good compatibility between the two materials promoted hydrogen bond formation between the PBS and LPRs. Rheological data showed good interfacial bonding between the materials, and the modulus of the high-melting PBS made the LPRs more damage resistant. When PBS was added at 30%, the tensile strength of the LPRs was increased by 2.8 times to 1.65 MPa, and the elongation at break increased by 31 times to 93%. This work demonstrates the potential of lignin thermoplastic phenolic resins for industrial applications and provides novel concepts for toughening biobased aromatic resins with PBS.

## 1. Introduction

Lignin is one of the most abundant natural polymers. It accounts for 15–40% of the dry weight in most plants, and its content is second only to that of cellulose [1]. An annual global production of over 70 million tons makes lignin the largest renewable source among aromatic biopolymers on the planet. Lignin is an amorphous crosslinked polymer consisting of benzene and propane units linked by carbon-carbon and ether bonds.

Because of its extremely complex structure, high resistance to biotransformation, and complex transformation products, lignin is difficult to utilize efficiently and is often used as a fuel for combustion in the pulp and paper production industry [2,3,4]. The waste generated by burning lignin as a fuel not only pollutes the environment but also causes a huge waste of resources. Lignin contains phenolic groups, which are capable of hydroxymethylation and polymerization upon condensation with formaldehyde, making it a prime candidate for replacement of petroleum-based phenol; lignin has ample potential in replacing phenol in the syntheses of high-value lignin phenolic resins [5]. Previous research on lignin phenolic resins has mostly been focused on phenolic foams, adhesives, and other thermosetting resins. The poor processability of the resin limits its applicability [6,7,8,9].

In contrast, the lignin phenolic resin obtained by acid-catalyzed reactions is thermoplastic and therefore has excellent processing properties, which can substantially increase its application scope and economic value. Our laboratory prepared thermoplastic, boron-modified highly adjacent phenolic resins using lignin, phenol, and formaldehyde as the raw materials and zinc acetate and oxalic acid as the catalysts under atmospheric pressure; this process improved the structure of the resin and enhanced the thermal stability and mechanical properties of the fibers by introducing boron into the molecular chains [10]. The effects of the acid ratio in the curing bath on the mechanical properties, thermal properties, and morphologies of the lignin phenolic fibers were also investigated, and the curing behavior of the lignin phenolic fibers was studied [11]. The lignin-based highly adjacent thermoplastic phenolic resin was produced by the partial substitution of phenol with enzymatic lignin, and then the lignin-based phenolic fibers were prepared by melt spinning. Lignin-based phenolic fibers with 10% lignin addition were found to have a good spinnability tensile strength (160.9 MPa) and elongation at break (1.9%), and the added lignin effectively improved the thermal properties of the fibers [12]. In our research on lignin phenolic resins, we found that due to the complex structure of lignin, synthetic lignin thermoplastic phenolic resins were not very tough, which greatly limited their application. Phenolic resin has a wide range of applications, such as the preparation of expanded graphite/phenolic resin composite bipolar plates with different phenolic resin contents by resin vacuum impregnation and hot-pressing methods [13]. Different formulations of phenolic resins based on two natural products, lignin and tannin, are used as wood bio-fireproof coatings [14]. A composite material with wear resistance and thermal conductivity was fabricated using phenolic resin and slab powder [15]. Due to the wide range of applications of phenolic resins, we have toughened and modified lignin phenolic resins to improve their performance and better apply them to various industries.

Phenolic resin toughening methods can be divided into chemically reactive toughening and physically commingled toughening. Chemical toughening involves a chemical reaction used to introduce flexible chain segments or graft flexible side chains onto the molecular chains of the phenolic resin to achieve the toughening effect. Commonly used chemical toughening agents include cashew phenol, polyurethane prepolymer, polyvinyl acetal, and carboxylated nitrile rubber [16,17]. Improving the molecular structure is the most direct solution, but there are problems of high costs and long development cycles. Physical toughening adds a nonreactive toughening agent to the phenolic resin by physical blending. Physical toughening is mainly based on the flexibility of the long chain of the toughening agent itself, the size effects of the microparticles, and the different dispersion forms of the toughening system in the phenolic resin, which inhibit crack expansion and absorb the stress on the phenolic cured products to achieve the toughening effect. Commonly used physical toughening agents are thermoplastic resins, rubber elastomers, carbon nanotubes, nano-SiO_2_, and other materials [18,19]. The advantages of a simple physical blending-type toughening method are low cost and the ability to achieve complementary properties when different polymer materials are mixed. There are many reports on the use of lignin to make phenolic resins. For example, the properties of thermosetting or thermoplastic phenolic resins prepared by different lignin modification methods were compared, and future research directions of synthetic biomass-based phenolic resins were proposed [20]. Lignin phenol formaldehyde resols have also been produced using depolymerized lignin fractions at various levels of phenol substitution (50 to 70 wt%). In another study, researchers liquefied corn stover lignin in hot-compressed water without any other additives and used it as a substitute for phenol to modify phenolic resins [1]. Researchers have also precipitated and isolated an industrial lignin from a sugarcane bagasse slurry that allowed the production of resins meeting standard requirements without modification or additives [21]. Phenolic resins have also been prepared from commercial kraft paper lignin [22]. As seen above, the current use of lignin for the manufacture of phenolic resins generally involves the modification of lignin followed by the synthesis of lignin phenolic resins or the use of lignin extracted from different biomass materials for the manufacture of lignin phenolic resins. Additionally, the preparation of LPRs has been rarely reported. No modification of LPRs using PBS has been reported.

Polybutylene succinate (PBS) is an aliphatic polyester with excellent ductility at room temperature, good melt processability, and excellent mechanical properties. PBS is very similar to the widely used polyethylene and polypropylene, thus allowing various processing methods, such as injection molding and film extrusion [23]. PBS is widely used in packaging, agriculture, medical supplies, and other fields [24,25]. PBS is also a biodegradable polymeric material that decomposes into CO_2_ and H_2_O in nature and is considered a green material. In summary, PBS has good melt processing ability and excellent mechanical properties. PBS is rich in polar groups such as hydroxyl and carbonyl groups and can be blended with polar lignin phenolic resins. Additionally, the hydroxyl groups in lignin thermoplastic phenolic resins (LPRs) provide hydrogen donors that form hydrogen bonds with polymers containing hydroxyl, carbonyl, ether, or other proton-accepting chain substituents [26]. PBS has hydroxyl and carbonyl groups that form hydrogen bonds with LPRs, thus introducing small flexible chain segments of PBS in the LPRs that act as toughening agents. Therefore, it is feasible to toughen the lignin phenolic resin by using PBS.

**Figure 1:**
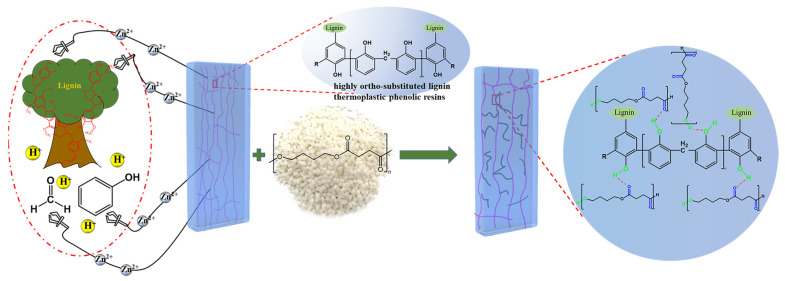
Modulation of highly ortho-substituted lignin thermoplastic phenolic resins and the PBS toughening mechanism.

Based on the above problems, we synthesized thermoplastic lignin phenolic resins by using lignin, phenol, and formaldehyde as raw materials. To address the brittleness and fragility of the lignin thermoplastic phenolic resin, we modified the resin with PBS for toughening (Figure 1). We analyzed the chemical bonding, mechanical properties, rheological properties, and thermal stabilities of the resins and modified resins. Using these analyses, we investigated the effects of different PBS additions on the physicochemical properties of the LPRs and discussed the molecular mechanism for PBS modification of the LPRs. The toughness of PBS-modified LPRs was substantially improved, and the homogeneities were enhanced. This study provides a novel concept for performance enhancement of lignin thermoplastic phenolic resins, which has important industrial application prospects of biobased polymeric materials.

## 2. Results

### 2.1. Synthesis of Lignin Highly Adjacent Thermoplastic Phenolic Resins

**Figure 2:**
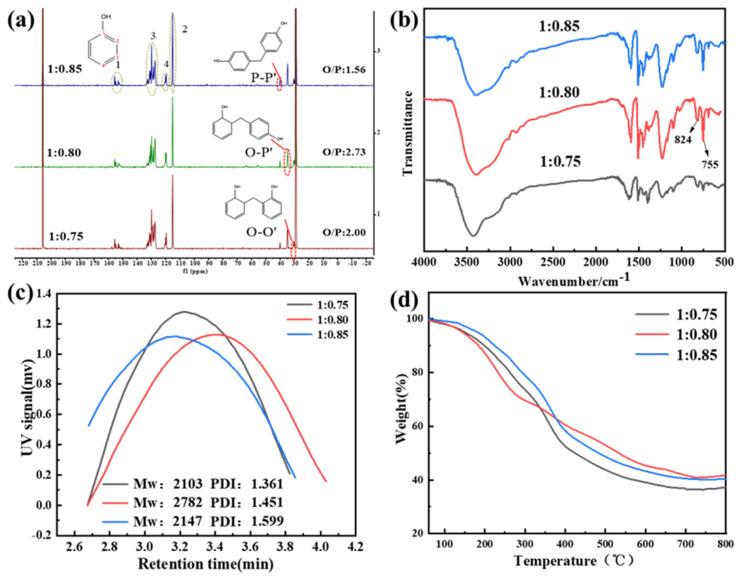
Performance analyses of the LPRs. (**a**) ^13^C-NMR profiles with different phenolic ratios, (**b**) FTIR profiles with different phenolic ratios, (**c**) GPC profiles with different phenolic ratios, and (**d**) TGA profiles with different phenolic ratios.

To obtain LPRs with higher molecular weights and high ortho ratios, the properties of the LPRs were tuned by changing the molar ratio of phenol and formaldehyde. The phenol–formaldehyde molar ratios were set to 1:0.75, 1:0.8, and 1:0.85, and the phenolic resins were prepared by using the same temperature and process. We performed NMR, FTIR, TGA, and GPC characterizations of the synthesized resins, as shown in Figure 2. The methylenes (-CH_2_-) shown in Figure 2a formed three types of linkages. The signals representing the methylene bonds (-CH_2_-), corresponding to ortho–ortho (O–O’), ortho–para (O–P’), or para–para (P–P’) connections within the phenolic resin, were observed at 31, 35.5, and 40 ppm, respectively. These can be used to calculate the resin neighbor/parity (*O*/*P*) values, as shown in Equation (1) [12].

(1) $$O/P=\frac{2AO–O+AO–P}{2AP–P+AO–P}$$

AP–P’, AO–P’, and AO–O’ are the signal strengths indicating the P–P’, O–P’, and O–O’ bonds, respectively. The calculated *O*/*P* values were 2.00, 2.73, and 1.56, respectively, and the highest value for the neighbor-to-neighbor ratio was found for the molar ratio 1:0.8, indicating that more regular LPRs were obtained with this molar ratio. To verify this result from the NMR analyses, FTIR studies of the resin were performed, as shown in Figure 2b. In the FTIR spectrum, the absorption peak observed at 824 cm^−1^ represents the para-substituted benzene ring, and the peak at 755 cm^−1^ represents the ortho-substituted benzene ring. We integrated the areas of these two peaks for the 1:0.75, 1:0.8, and 1:0.85 LPRs and then performed a semiquantitative analysis. The ratios were 1.49, 1.93, and 1.63, respectively, which showed that the highest *O*/*P* value was that for the LPR prepared with a 1:0.8 molar ratio; this was the same result obtained with NMR analyses. Figure 2c shows the weight average molecular weights (Mw) and molecular weight distributions (PDI) of the three resins. Figure 2d summarizes the thermal performance indexes of the three resins. Excessively high phenolic ratios destabilized the reaction system, leading to a lower degree of polymerization and a decrease in the neighbor-to-pair ratio and resin performance. In general, the biobased highly adjacent phenolic resins synthesized from phenol, formaldehyde (the phenol/formaldehyde ratio was 1:0.80), and lignin with double catalysis by zinc acetate and oxalic acid had *O*/*P* values up to 2.73, molecular weights up to 1917 g/mol, molecular weight distributions of 1.451, and residual carbon proportions of 41.7% after heating at 800 °C, with excellent heat resistance. Based on the above analysis, the lignin phenolic resin synthesized with a phenol–formaldehyde ratio of 1:0.80 exhibited a better overall performance.

### 2.2. Fourier Transform Infrared Spectroscopy (FTIR) Analyses and X-ray Diffraction (XRD) Analyses

**Figure 3:**
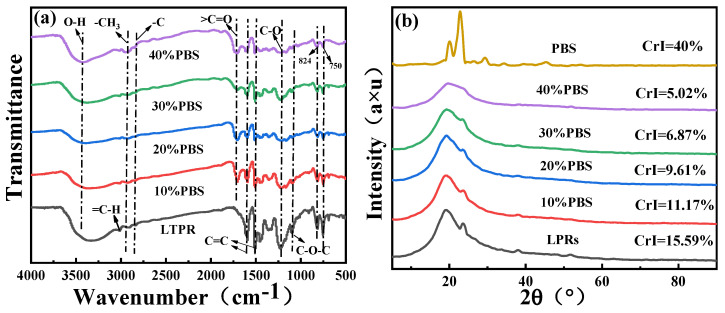
(**a**) FTIR spectra. (**b**) XRD spectra.

LPRs are brittle and exhibit poor mechanical properties due to their structures. For this reason, we used PBS to toughen and modify LPRs and then analyzed their properties and the toughening mechanism. We doped LPRs with 10%, 20%, 30%, and 40% PBS to determine the optimal modification ratio. Figure 3a shows the FTIR spectra of the blended resins. In Figure 3a, the O-H stretching vibrational band for the phenolic hydroxyl groups near 3320 cm^−1^ also corresponded to the hydroxyl groups of the LPRs due to hydrogen bonding self-conjugation. With the addition of PBS, the O-H band shifted to approximately 3420 cm^−1^, indicating that a competition between the hydroxyl/hydroxyl and hydroxyl/carbonyl interactions produced a new hydrogen bond distribution, and most of the self-conjugated hydrogen bonds broke to form mutually bonded hydrogen bonds [27]. Therefore, the hydroxyl groups of the LPRs underwent hydrogen bonding with the carbonyl, hydroxyl, and ether bonds of PBS. The peak at 3020 cm^−1^ corresponded to a (=C-H) stretching vibration of the arene rings [28], and the symmetric stretch of the methyl group (-CH_3_) and the asymmetric stretch of the methylene group (-C) produced bands at 2950 cm^−1^ and 2830 cm^−1^, respectively. The 1710 cm^−1^ ester carbonyl (C=O) absorption peak appeared for the resin after the addition of PBS, but the 1720 cm^−1^ peak shifted slightly toward lower wavenumbers relative to the ester carbonyls (C=O) of the PBS. This indicated that hydrogen bonding occurred between the ester carbonyls (C=O) of the PBS and the OH groups in the LPRs [29]. The peaks at 1600 cm^−1^ and 1510 cm^−1^ were C=C stretching vibrations specific to the benzene ring backbone [30]; the peaks at approximately 1230 cm^−1^ and 1100 cm^−1^ were for C-O stretching vibrations on the benzene rings and the asymmetric stretching vibration of the nearby ether bonds (C-O-C), respectively. The peaks at approximately 824 cm^−1^ and 750 cm^−1^ corresponded to para-substitution of the phenolic hydroxyl groups of the benzene ring and the neighboring substitution of the cyclic phenolic hydroxyl groups, respectively [12]. This analysis of infrared spectra showed that PBS modified the LPRs by physical blending and also by generating many hydrogen bonds between the molecular chains of the two materials, which reduced the interfacial energy between the phases, improved the interfacial adhesion, and increased its interfacial compatibility.

Figure 3b shows the X-ray spectra of the blended resins; the X-ray spectra of the LPRs showed two major crystallographic diffraction peaks at *2θ* values of 18.9° and 23.7°, which were attributed to the (203) and (220) crystal planes, respectively [31]. The two main diffraction peaks for pure PBS appeared at *2θ* values of 19.9° and 22.9° and were attributed to the (020) and (110) crystal planes, respectively [32]. In general, a higher crystallinity corresponds to a less brittle and ductile polymer. The intensities of the two crystalline diffraction peaks decreased with increasing PBS content; therefore, the crystallinity also decreased with increasing PBS content, as shown by the XRD patterns. The addition of PBS may have broken the regular arrangements of the LPR molecular chain segments, thus enhancing the toughness of the resin. The analysis showed that this was potentially due to embedding of the small flexible PBS chain segments within the regular long LPR chain segments, which increased their toughness.

### 2.3. Thermogravimetric Analyses (TGA-DTG)

**Figure 4:**
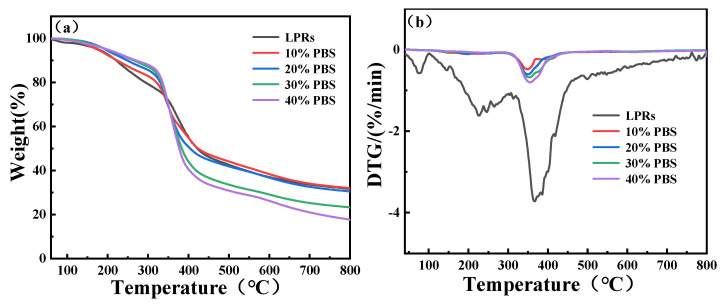
TGA-DTG curves; (**a**) TGA; (**b**) DTG.

Figure 4 shows the TGA-DTG curves of the LPRs and the co-blended resins, and Table 1 shows the TGA-DTG data. As shown in Figure 4, the LPRs and PBS-modified LPRs exhibited continuous mass losses with increasing temperature, and there were mainly three stages of mass loss. In the first stage, which occurred between 0 °C and 300 °C, the mass loss mainly involved evaporation of residual water from inside the resin, further condensation reactions of the LPR to remove water, and decomposition of some small phenolic oligomers. In the second stage, between 300 °C and 450 °C, mass losses were attributed to oxidation of the methylene groups attached to the aromatic rings and the onset of resin decomposition into hydrogen, carbon dioxide, water, and other small molecules. At this stage, the highest mass loss was observed, and the rate of mass loss increased significantly after PBS modification of the LPRs. In the third stage, occurring between 450 °C and 800 °C, the resin began to carbonize, and the PBS was completely decomposed into CO_2_ and H_2_O; the amount of residual mass decreased with increasing PBS content in the original sample.

As shown by the TGA-DTG data in Table 1, the first weight losses of the LPRs and PBS-modified LPRs between 0 °C and 300 °C were 20.8%, 17.1%, 14.4%, 12.9%, and 12% for the LPR and the LPRs containing 10% PBS, 20% PBS, 30% PBS, and 40% PBS, respectively. The weight losses decreased with increasing PBS content, indicating that blending of the LPRs with PBS in the lower temperature range improved the thermal stability of the blended resin due to the higher thermal stability of PBS up to 300 °C and additional mass losses from dehydration polymerization of the LPRs [33]. The second temperature range for weight loss was 300 °C to 800 °C. In this temperature range, the weight losses of the LPR for 10% PBS, 20% PBS, 30% PBS, and 40% PBS resins were 47.5%, 51%, 55.2%, 63.9%, and 70.47%, respectively. The weight losses increased with increasing PBS content, indicating that the maximum temperature for thermal stability of the LPRs blended with the PBS resin decreased rapidly. PBS was not resistant to high temperatures and decomposed quickly into CO_2_ and H_2_O. In general, the added PBS improved the low-temperature thermal stability. Figure 4b shows a single peak in the DTG plot of the composite resin, indicating that the added PBS improved the homogeneity of the resin. These results combined with the FTIR and XRD data showed that a more compatible structure was formed between the two materials, and the homogeneities of the composites were better than that of the LPR.

### 2.4. Differential Scanning Calorimetry (DSC) Analyses

**Figure 5:**
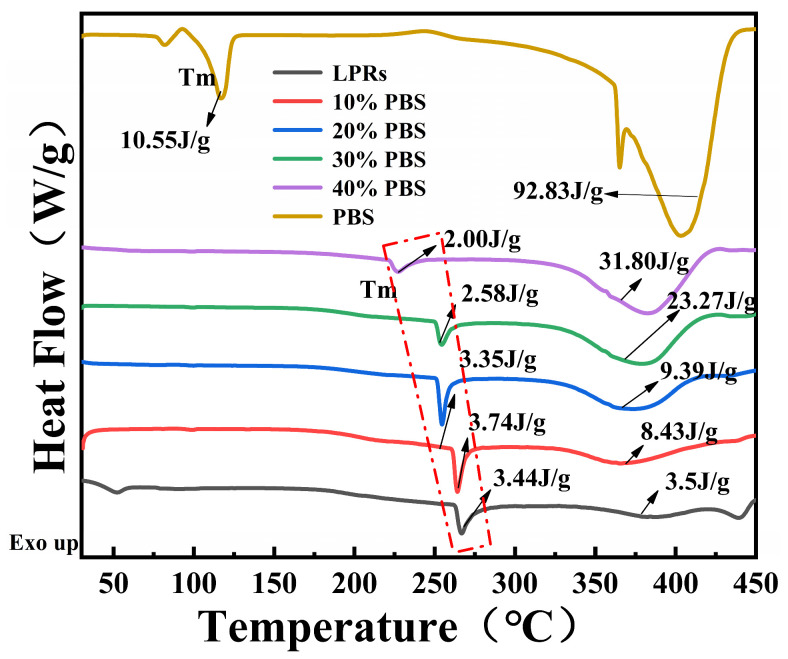
DSC curves.

Figure 5 shows the DSC curves for the LPRs and the blended resins; sharp endothermic peaks at 267 °C and 384 °C for the melting peak (T_m_) were observed for the LPRs. Based on the TGA data, the endothermic peaks appearing at 267 °C and 384 °C were due to LPR melting, which requires heat absorption, and the heat absorbed by decomposition, respectively. The endothermic peaks for PBS appeared at 116 °C and 404 °C for melting and decomposition, respectively. The phase changes of PBS-modified LPRs appeared near 260 °C, and the T_m_ decreased with increasing PBS addition. The enthalpy (ΔHm) also decreased with increasing PBS content; combined with the XRD data, this indicated that the crystallinity of the molecular chain segments were decreased by the addition of PBS, the orientation of the chain segments decreased, and thus, the energy required for disordering of the chain segments during melting was reduced. The endothermic peaks corresponding to degradation of the LPRs modified with PBS were located at approximately 384 °C. The enthalpy of degradation increased with increasing PBS addition and shifted to higher temperatures close to the thermal degradation peak of PBS at 404 °C. This indicated that the homogeneities of the composites were significantly improved, which was consistent with the DTG data. Upon addition of the PBS, the PBS melting peak at 116 °C did not appear for the blended resin, indicating that LPRs and PBS underwent molecular level commingling during processing and there was no agglomerated PBS; therefore, the composites exhibited good compatibility [34].

### 2.5. Rheological Performance Analyses

**Figure 6:**
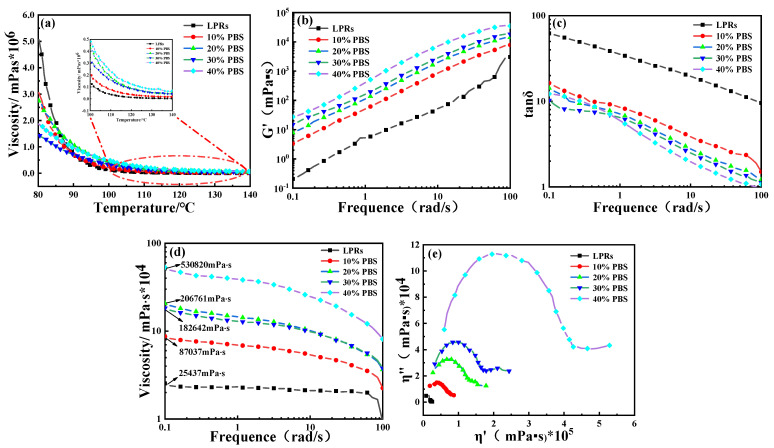
(**a**) Plots of viscosity versus temperature; (**b**) plots of G’ versus frequency; (**c**) plots of loss factor versus frequency; (**d**) dynamic viscosity curves; and (**e**) Cole–Cole plots of virtual viscosity (η″) versus real viscosity (η’).

Figure 6a shows the viscosity curves for the LPR and blended resins as a function of temperature. As seen in Figure 6a, the viscosities of pure LPRs were higher than those of the blended resins at lower temperatures; the viscosities of the LPRs up to 80 °C were much higher than those of the blended resins. The viscosities of the LPRs dropped quickly above 90 °C, indicating higher viscosities of the blended resins relative to those of the LPRs; the resin viscosities increased as the amount of PBS added increased. The changes in viscosity showed that the LPR viscosity was too high at low temperatures (80 °C to 90 °C), which inhibited plasticization of the material. The addition of PBS reduced the low-temperature viscosity and improved the processability. Above 100 °C, the viscosities of the PBS-containing samples increased with increasing temperature. Therefore, addition of PBS broadened the processing temperature range of the LPR, which enabled molding of the LPR.

Figure 6b shows a plot of the storage modulus (*G*’) versus frequency. *G*’ is a measure of the energy stored in the material and depends on the relative number of molecular chain rearrangements that can occur during the oscillation period and is used to estimate the elasticity of the material [35]. The energy storage moduli of the blended resins increased with increasing PBS content, as shown in Figure 6b. The analysis showed that the continuous structure and enhanced interfacial interactions between the different components also contributed to the increased modulus of the polymer melt, especially in the low frequency range [36]. Therefore, PBS was more compatible with the LPRs in co-continuous structures. If *G*’ fluctuates, the material is less compatible and stable. These fluctuations are caused by two possible factors. First, in immiscible binary polymer blends, the interface between the two phases is deformed and relaxed during the rheological measurements. The other factor involves fluctuations of the concentration in the mix. Near the phase separation point, the magnitude of this fluctuation usually becomes larger, and the fluctuation time is longer. When the viscoelastic properties of the two components are very different, concentration fluctuations can lead to large dynamic heterogeneity of the system. If the applied flow fails to dissipate the fluctuations, the resulting rheological data become similar to the case of a heterogeneous system without an interface [37]. The *G*’ values for the PBS-modified resins did not fluctuate at low frequencies; therefore, after adding PBS to the modified LPRs, the blended resin was very compatible, and no phase separation occurred.

Figure 6c shows the loss factor (tan δ) versus frequency. The loss factor is calculated with the following equation: tan δ = *G*″/*G*’. The size of the loss factor represents the viscoelastic properties of the material; a larger loss factor indicates a greater viscosity of the material, and a smaller loss factor indicates a greater elasticity of the material. The loss factor of the PBS-modified resin was much lower than that of the LPR, as seen in Figure 6c, indicating that the co-blended resin was more elastic and resistant to damage at high frequencies.

Figure 6d shows the dynamic viscosity curves, and the zero-shear viscosity (η_0_) of the LPRs was much lower than those of PBS-modified LPRs. Increases in the values of η_0_ were observed with increasing PBS content. The shear viscosity of a polymer is a very important indicator for the production and processing methods. Viscous flow of a polymer indicates synergistic movement of individual polymer chains along the direction of force. Therefore, a more severe entanglement between the polymer molecular chains and higher friction between the molecular chains generate a higher resistance to motion of the polymer chain segments and a higher shear viscosity [38]. Therefore, the modified LPRs exhibited better compatibility. As shown in Figure 6d, the shear viscosities of the pure LPRs and the blended resins had minimal dependence on low and medium frequencies, indicating Newtonian fluid properties, whereas in the high frequency region, the shear viscosities decreased with increasing frequency, indicating shear thinning behavior [39]. The shear viscosities of pure LPRs were low; however, the shear viscosities of the blended resins were increased by addition of PBS, and the shear viscosities increased with increasing PBS content. With addition of PBS, the flexible chain segments of PBS entangled with the long chain segments of LPRs. This also indicated that the PBS chain segments entered into the long chain segments of LPRs, thus providing a toughening effect.

Figure 6e shows the Cole–Cole plots of the virtual viscosity (η″) and real viscosity (η’). Since immiscible mixtures with different phase morphologies exhibit different relaxation mechanisms, the Cole–Cole diagrams describing the relationship between the imaginary part η″ and the real part η’ of the complex viscosity should show different shapes [40]. For immiscible blends, the Cole–Cole plots of η″ and η’ produce two arcs, which are related to the appearance of two processes with different relaxation times. As seen in Figure 6e for PBS-modified LPRs, only one arc was observed. This showed that PBS was compatible with the LPRs, and the LPRs and PBS blending system exhibited good interfacial bonding.

### 2.6. Scanning Electron Microscopy (SEM)

**Figure 7:**
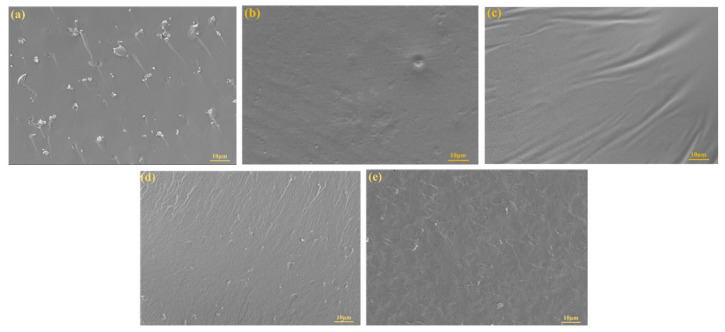
SEM images; (**a**) LPR; (**b**) 10% PBS addition; (**c**) 20% PBS addition; (**d**) 30% PBS addition; and (**e**) 40% PBS addition.

The micromorphology of a blend substantially contributes to its mechanical properties. Figure 7 shows SEM images of low-temperature fracture surfaces of PBS-modified LPRs with different contents. Some of the factors affecting the morphologies of immiscible blends are the composition, processing conditions, viscosity ratio of the components, and interfacial tension. Immiscible blends usually exhibit a “sea-island” phase structure indicating the structural characteristics of phase separation and a matrix–droplet morphology. As seen in Figure 7, after addition of PBS, the cross section did not show the “sea-island” phase structure, the phase separation structure, or the matrix–droplet morphology [41]. This indicates that LPRs were very compatible with PBS and were miscible. The impact section of pure LPRs was relatively smooth, indicating that pure LPRs were less ductile and prone to brittle fracture [42,43]. The surface of the pure LPRs contained some small particles, which were small lignin particles that were not fully liquefied. With increasing PBS content, the fracture surfaces of the small particles decreased, and the fracture surface became rough above 20% PBS content, which showed a toughness fracture. This indicated that PBS addition increased the toughness of the LPRs very clearly.

### 2.7. Mechanical Analyses

**Figure 8:**
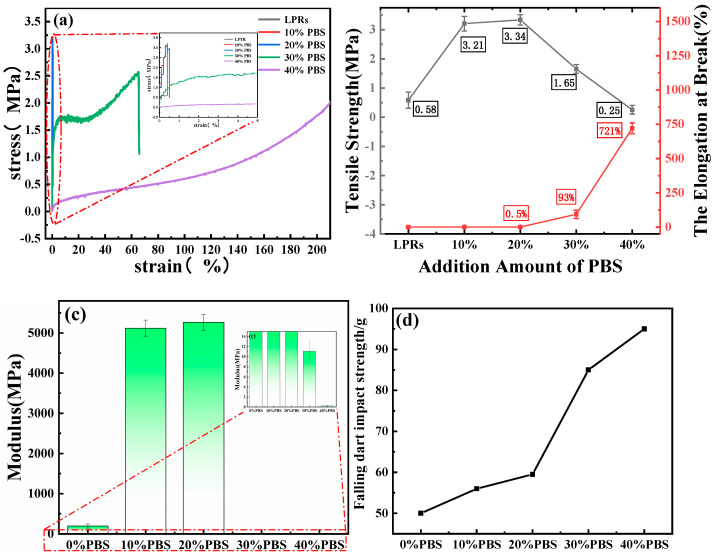
(**a**) Stress-strain diagram; (**b**) tensile strength vs. elongation at break; (**c**) tensile modulus; and (**d**) falling dart impact strength.

Figure 8a shows the stress–strain diagram of the resin. Pure LPRs are hard and brittle resins, as shown in Figure 8a. There was no yield inflection point in the stress–strain curve, which is typical of brittle fracture [44]. The tensile strength was 0.5 MPa, and the elongation at break was 0.3%. When the PBS content was 10% or 20%, the tensile strength and elongation at break of the blended resin increased with increasing PBS content. The yield inflection point occurred when the PBS content was increased to 30%; the yield strength was 2.57 MPa, the tensile strength decreased, and the elongation at break rapidly increased. When the amount of PBS added was 40%, the tensile strength rapidly decreased, and the elongation at break rose sharply and was difficult to measure, indicating soft characteristics.

Figure 8b shows a plot of tensile strength versus elongation at break, and the tensile strength of the blended resin was significantly enhanced by the addition of PBS in the range 10–30%; the strength of the resin with 20% addition was increased to 3.34 MPa, and the strength started to decrease beyond 20%. The elongation at break increased with increasing PBS, with an important threshold value of 20% and a significant increase in elongation at break, up to 721%, above 20% PBS content. The addition of PBS below 30% significantly improved both the toughness and the tensile strength of the LPRs. The tensile strength was improved (1.65 MPa), and the elongation at break was significantly increased (93%) when the amount of PBS added reached 30%, which ensured that there was an excellent tensile resistance background and enhanced the strength of the resin. The analysis showed that PBS could be homogeneously blended with LPRs with effective entanglement of the molecular chains, and the FTIR data showed the formation of hydrogen bonds between the materials. This greatly contributed to the performance improvement of LPRs.

Figure 8c shows the tensile modulus map. Tensile modulus refers to the elasticity of a material when it is stretched. Its value is the ratio of the force required to stretch the material per unit length in the central axis direction to the cross-sectional area. It can be seen from the figure that the tensile moduli of 0% PBS, 10% PBS, 20% PBS, 30% PBS, and 40% PBS were 194 MPa, 5121 MPa, 5264 MPa, 11 MPa, and 0.24 MPa, respectively. The tensile modulus increases first and then decreases with the increase of PBS content. When the content of PBS was lower than 20%, the tensile modulus was very large, and it reached a maximum at 20% PBS, which is due to the low elongation at break. When the content of PBS reached 30%, the tensile modulus decreased because the elongation at break increased significantly. It can be seen from Figure 8b that when the addition amount was less than 20%, the rigidity of the blend resin was very strong and showed a very brittle property. When the addition amount reached 30%, the rigidity of the blend resin decreased, showing a certain degree of rigidity and toughness.

Figure 8d shows the impact strength map of falling darts. From the figure, we can see that the impact strength of the blended resin increased with an increase in the amount of PBS added. The impact strength started to increase rapidly from 20% PBS, and it increased by 70% compared to the impact strength of the LPRs without PBS when the PBS addition reached 30%, which corresponds to a rapid increase in elongation at break starting from 20%. This further demonstrates that the addition of PBS does indeed have a toughening effect on LPRs.

## 3. Discussion

In this study, LPRs were synthesized by partial substitution of phenol with lignin, and a lignin phenolic resin with an *O*/*P* ratio of 2.73 was synthesized by modulating the molar ratio of Zn^2+^ ions and phenolic aldehydes. To address the problem of poor toughness in the highly adjacent LPRs, LPRs were modified with different doping amounts of PBS, and then the co-modified resins were analyzed and mechanically evaluated. From the DSC, it can be seen that the melting peak of PBS at 116 °C did not appear after the addition of PBS, while DTG only showed the maximum pyrolysis peak at 350 °C, which indicates that the addition of PBS effectively improved the homogeneity of the material. Additionally, PBS effectively reduced the melting temperature and melt enthalpy of the LPRs, which enabled molding of the material. The addition of PBS improved the elastic moduli of the LPRs, reduced their loss factors, and improved their impact resistances under high frequency. The PBS molecular chains were entangled with the LPR chains at the molecular level and commingled uniformly, effectively breaking their crystallization, decreasing the crystallinity, and increasing the toughness. The 30% PBS-modified LPR had better toughness, an elongation at break of 93%, and a better strength of 1.65 MPa. In general, we prepared a highly adjacent phenolic resin using lignin. These LPRs were modified and reinforced with PBS to create biobased resins. A novel concept for materialization of biomass aromatic compounds and performance enhancements was provided.

At present, there are two common ways to manufacture lignin-based phenolic resins with different properties. One is to select different biomass resources to produce different lignin structures, and the other is to make different lignin-based phenolic resins with different properties through different modifications of lignin. To summarize, the prepared LPRs were modified by physical melt blending. This provides a simple and low-cost method to increase the strength of lignin phenolic resin. At the same time, it also proposes a new method to improve the performance of biomass aromatic compounds.

## 4. Materials and Methods

### 4.1. Materials

Colorless crystalline phenol, AR grade, was obtained from Guangdong Guanghua Technology Co., Ltd. (Shantou, China). A formaldehyde solution, which was acquired from Chengdu Jinshan Chemical Reagent Co., Ltd. (Chengdu, China), was a colorless, transparent liquid with a concentration of 37–40%. White flake or granular crystals of zinc acetate, AR grade, were obtained from Tianjin Fengchuan Chemical Reagent Technology Co., Ltd. (Tianjin, China). Oxalic acid, colorless crystals, was purchased from Tianjin Fengchuan Chemical Reagent Technology Co., Ltd., AR grade. Enzymatic straw lignin, brown powder, with a lignin content ≥ 80% was purchased from Shandong Longli Biotechnology Co., Ltd. (Yucheng, China). Polybutylene succinate, a white semicrystalline polymer, was purchased from Lanshan Tunhe Technology Co., Ltd. (Changji, China).

### 4.2. Methods

A Fourier transform infrared spectrometer (FTIR) (Waltham, MA, USA) with a scan range of 4000 cm^−1^ to 400 cm^−1^ was used in this study, and 64 scans were collected to examine the interactions between PBS and LPRs with the potassium bromide compression method (powder size below 2 μm). ^13^C-NMR spectroscopy was performed using an ECZ 400S NMR instrument (JEOL, Tokyo, Japan). The 50 mg samples were dissolved in deuterated acetone. The methyl peak of deuterated acetone was set to 29 ppm. The resin was ground into 2 μm fragments. An X-ray diffractometer (XRD), model max220 (Neo-D, Japan), with Cu Kα radiation (λ = 1.5406 nm) was used to collect diffraction data, and the relative crystallinities were analyzed with Jade 6 software (International Centre for Diffraction Data, Newtown, PA, USA). A TG 209 F3 Tarsus (NETZSCH, Selb, Germany) was used for TG-DTG studies. Samples weighing 10 mg were heated at a rate of 10 °C/min over the temperature range 30–800 °C and were protected by nitrogen with a flow rate of 20 mL/min. Specimens weighing 5–10 mg were heated from 30 °C to 450 °C with a NETZSCH CC 200 differential volume scanning calorimeter (DSC) (NETZSCH, Selb, Germany) under a N_2_ atmosphere with a temperature increase rate of 10 °C/min. Rheological studies were carried out using a Haake Mars iQ rotating rheometer (Thermo Fisher Scientific, Waltham, MA, USA). For the temperature scans, the temperature range was 80–140 °C, the temperature interval was 5 °C/min, the frequency was set to 1 rad/s, and the set strain control was 10% to scan the temperature of the sample. For the frequency scans, the strain control was set to 10%, and frequency scans of the sample were run from 0.1 rad/s to 100 rad/s. The sample was given a gold spray treatment before being examined with an SU8010 scanning electron microscope (Hitachi, Tokyo, Japan) with a working distance of 8.5 mm and an accelerating voltage range of 0.02–30 kV. Analyses were performed on the resin surface morphologies. First, dumbbell-shaped specimens were fabricated according to the National Standard GB/T1040.2/1A/25 of the People’s Republic of China using a homemade mold and graphite heating platform. The tensile strength and elongation at break, two mechanical characteristics of the resin, were assessed with a PARAM XLW (PC) intelligent electronic tensile tester (Jinan Labthink Mechatronics Technology Co., Ltd., Jinan, China) with a stretching speed of 25 mm/min. The sample was tested using a BMC-B1 dart impact testing machine (Labthink Instruments Co., Ltd., Jinan, China) in accordance with the national standard GB/T 9639.1-2008 of the People’s Republic of China to evaluate the impact performance of the resin.

### 4.3. Preparation of the Lignin Phenolic Resin

Phenol (90 g), lignin (9 g), and zinc acetate (4.4 g) were added to a condenser-equipped single-necked round-bottom flask with an attached tube and a rotor and heated for 0.5 h at 120 °C to mix the phenol and lignin thoroughly and to liquefy the lignin. Then, the reaction temperature was lowered, and the formaldehyde solution with different molar ratios was introduced and reacted for 2 h. After that, the mixture was heated to 100 °C and maintained there for two hours after the addition of oxalic acid. After the reaction, the mixture was dissolved in ethanol and distilled under reduced pressure to eliminate unreacted phenol and water to produce a lignin-based thermoplastic phenolic resin.

### 4.4. Preparation of the Polybutylene Succinate-Modified Resin

Polybutylene succinate was added to the lignin phenolic resin in proportions of 10%, 20%, 30%, and 40% of the total amount, and then the material was scraped out and loaded on a tray after 30 min of refining at 120 °C with a speed of 20 r/min in a dense refiner.

## Figures and Tables

**Table 1:** TGA-DTG data for the LPR and blended samples.

Samples	Maximum Weight Loss Rate Temperature/°C	Maximum Weight Loss Rate/% × min^−1^	Weight Losses in Different Temperature Ranges (%)		Residual Mass/%
			0–300 °C	300–800 °C	
LPR	367	3.7	20.8	47.5	31.7
10% PBS	350	0.48	17.1	51	31.9
20% PBS	353	0.61	14.4	55.2	30.4
30% PBS	356	0.68	12.9	63.9	23.2
40% PBS	356	0.8	12	70.4	17.6

## Data Availability

The data presented in this study are available on request from the corresponding author.

## References

[1] Yan L.C., Cui Y.H., Gou G.J. (2017). Liquefaction of lignin in hot-compressed water to phenolic feedstock for the synthesis of phenol-formaldehyde resins. Compos. Part B-Eng..

[2] Rodrigues J.S., de Freitas A.S.M., Maciel C.C. (2023). Selection of kraft lignin fractions as a partial substitute for phenol in synthesis of phenolic resins: Structure-property correlation. Ind. Crops Prod..

[3] Cao Y., Chen S.S., Zhang S. (2019). Advances in lignin valorization towards bio-based chemicals and fuels: Lignin biorefinery. Bioresour. Technol..

[4] Wang M., Wang F. (2019). Catalytic scissoring of lignin into aryl monomers. Adv. Mater..

[5] Sternberg J., Sequerth O., Pilla S. (2021). Green chemistry design in polymers derived from lignin: Review and perspective. Prog. Polym. Sci..

[6] Melro E., Antunes F.E., Valente A.J.M. (2022). On the development of phenol-formaldehyde resins using a new type of lignin extracted from pine wood with a levulinic-acid based solvent. Molecules.

[7] Gao C., Li M., Zhu C. (2021). One-pot depolymerization, demethylation and phenolation of lignin catalyzed by HBr under microwave irradiation for phenolic foam preparation. Compos. Part B-Eng..

[8] Li B., Wang Y., Mahmood N. (2017). Preparation of bio-based phenol formaldehyde foams using depolymerized hydrolysis lignin. Ind. Crops Prod..

[9] Yang W., Rallini M., Natali M. (2019). Preparation and properties of adhesives based on phenolic resin containing lignin micro and nanoparticles: A comparative study. Mater. Des..

[10] Ren Y., Lin X., Shi Z. (2021). Improving the thermal and mechanical properties of phenolic fiber over boron modified high-ortho phenolic resin. High Perform. Polym..

[11] Ren Y., Lin X., Wang W. (2021). Preparation of high molecular weight thermoplastic bio-based phenolic resin and fiber based on lignin liquefaction. Mater. Res. Express.

[12] Ren Y., Xie J., He X. (2021). Preparation of Lignin-Based High-Ortho Thermoplastic Phenolic Resins and Fibers. Molecules.

[13] Li W., Jing S., Wang S. (2016). Experimental investigation of expanded graphite/phenolic resin composite bipolar plate. Int. J. Hydrogen Energy.

[14] De Hoyos-Martinez P.L., Issaoui H., Herrera R. (2021). Wood Fireproofing coatings based on biobased phenolic resins. ACS Sustain. Chem. Eng..

[15] Binda F.F., de Alvarenga Oliveira V., Fortulan C.A. (2020). Friction elements based on phenolic resin and slate powder. J. Mater. Res. Technol..

[16] Tang K., Tang X., Liu X. (2022). Phenolic Foams Toughened with Triethylene Glycol by In Situ Polymerization and Prepolymerization Processes. ACS Appl. Polym. Mater..

[17] Yu Y., Wang Y., Xu P. (2018). Preparation and characterization of phenolic foam modified with bio-oil. Materials.

[18] Aliakbari M., Jazani O.M., Sohrabian M. (2019). Epoxy adhesives toughened with waste tire powder, nanoclay, and phenolic resin for metal-polymer lap-joint applications. Prog. Org. Coat..

[19] Xu P., Yu Y., Chang M. (2019). Preparation and characterization of bio-oil phenolic foam reinforced with montmorillonite. Polymers.

[20] Gao Z., Lang X., Chen S. (2021). Mini-review on the synthesis of lignin-based phenolic resin. Energy Fuels.

[21] Maree C., Görgens J.F., Tyhoda L. (2022). Lignin phenol formaldehyde resins synthesised using South African spent pulping liquor. Waste Biomass Valorization.

[22] Liu Q., Xu Y., Kong F. (2022). Synthesis of phenolic resins by substituting phenol with modified spruce kraft lignin. Wood Sci. Technol..

[23] Hu C., Bourbigot S., Delaunay T. (2019). Synthesis of isosorbide based flame retardants: Application for polybutylene succinate. Polym. Degrad. Stab..

[24] Hu C., Bourbigot S., Delaunay T. (2020). Poly (isosorbide carbonate): A ‘green’char forming agent in polybutylene succinate intumescent formulation. Compos. Part B-Eng..

[25] Vytejčková S., Vápenka L., Hradecký J. (2017). Testing of polybutylene succinate based films for poultry meat packaging. Polym. Test..

[26] Zhang Y., Zhang L., Zhang C. (2022). Continuous resin refilling and hydrogen bond synergistically assisted 3D structural color printing. Nat. Commun..

[27] Du W.T., Kuan Y.L., Kuo S.W. (2022). Intra-and Intermolecular Hydrogen Bonding in Miscible Blends of CO_2_/Epoxy Cyclohexene Copolymer with Poly (Vinyl Phenol). Int. J. Mol. Sci..

[28] Supthanyakul R., Kaabbuathong N., Chirachanchai S. (2016). Random poly (butylene succinate-co-lactic acid) as a multi-functional additive for miscibility, toughness, and clarity of PLA/PBS blends. Polymer.

[29] Sukhawipat N., Saengdee L., Pasetto P. (2022). *Caesalpinia sappan* L. wood fiber: Bio-reinforcement for polybutylene succinate-based biocomposite film. Cellulose.

[30] Kalami S., Arefmanesh M., Master E. (2017). Replacing 100% of phenol in phenolic adhesive formulations with lignin. J. Appl. Polym. Sci..

[31] Chen H.P., Nagarajan S., Woo E.M. (2020). Unusual Radiating-Stripe Morphology in Nonequimolar Mixtures of Poly (l-lactic acid) with Poly (d-lactic acid). Macromolecules.

[32] Zhang X., Shi J., Zhou J. (2022). Nucleation effect of cellulose nanocrystals/polybutylene succinate composite filler on polylactic acid/polybutylene succinate blends. Polym. Bull..

[33] Mohamad N., Mazlan M.M., Tawakkal I.S.M.A. (2022). Characterization of active polybutylene succinate films filled essential oils for food packaging application. J. Polym. Environ..

[34] Takhulee A., Takahashi Y., Vao-soongnern V. (2017). Molecular simulation and experimental studies of the miscibility of polylactic acid/polyethylene glycol blends. J. Polym. Res..

[35] Naito Y., Nishikawa M., Mobuchon C. (2021). Effect of rheological transitions in matrix resin on flow mechanism of carbon Fiber/Epoxy prepreg. Compos. Part A-Appl. Sci. Manuf..

[36] Wu F., Misra M., Mohanty A.K. (2020). Tailoring the toughness of sustainable polymer blends from biodegradable plastics via morphology transition observed by atomic force microscopy. Polym. Degrad. Stab..

[37] Takhulee A., Takahashi Y., Vao-soongnern V. (2017). Molecular simulation and experimental studies of the miscibility of PLA/PLA_x_-PEG_y_-PLA_x_ blends. J. Polym. Res..

[38] Mohapatra A.K., Mohanty S., Nayak S.K. (2016). Properties and characterization of biodegradable poly (lactic acid) (PLA)/poly (ethylene glycol) (PEG) and PLA/PEG/organoclay: A study of crystallization kinetics, rheology, and compostability. J. Thermoplast. Compos. Mater..

[39] Jariyasakoolroj P., Chirachanchai S. (2022). In Situ Chemical Modification of Thermoplastic Starch with Poly (l-lactide) and Poly (butylene succinate) for an Effectively Miscible Ternary Blend. Polymers.

[40] Jia S., Zhao L., Wang X. (2022). Poly (lactic acid) blends with excellent low temperature toughness: A comparative study on poly (lactic acid) blends with different toughening agents. Int. J. Biol. Macromol..

[41] Xiao H., Huang Z.X., Zhang Z.P. (2022). Highly thermally conductive flexible copper clad laminates based on sea-island structured boron nitride/polyimide composites. Compos. Sci. Technol..

[42] Mao Z., Zhang X., Jiang G. (2019). Fabricating sea-island structure and co-continuous structure in PMMA/ASA and PMMA/CPE blends: Correlation between impact property and phase morphology. Polym. Test..

[43] Li X., Luo X., Gu Y. (2015). A novel benzoxazine/cyanate ester blend with sea-island phase structures. Phys. Chem. Chem. Phys..

[44] Mukherji S., Kandula N., Sood A.K. (2019). Strength of mechanical memories is maximal at the yield point of a soft glass. Phys. Rev. Lett..

